# Cognitive function and cardiorespiratory fitness affect gait speed in type-2 diabetic patients without neuropathy

**DOI:** 10.3325/cmj.2022.63.544

**Published:** 2022-12

**Authors:** Gulin Findikoglu, Abdurrahim Altinkapak, Hakan Alkan, Necmettin Yildiz, Hande Senol, Fusun Ardic

**Affiliations:** 1 Department of Physical Medicine and Rehabilitation, Pamukkale University, Denizli, Turkey; 2 Department of Biostatistics, Pamukkale University, Denizli, Turkey

## Abstract

**Aim:**

To identify physical, cognitive, and metabolic factors affecting gait speed in patients with type-2 diabetes mellitus (T2DM) without neuropathy.

**Methods:**

This cross-sectional study enrolled 71 diabetic patients without neuropathy (mean age 55.87 ± 7.74 years, 85.9% women). Neuropathy status was assessed with Douleur Neuropathique 4. We used a cut-off point for gait speed of 1 m/s to classify the participants into two groups: slow walkers (SW) and average and brisk walkers (ABW). The groups were compared in terms of age, sex, body mass index (BMI), hemoglobin A1c (HbA1c), fasting glucose, systolic blood pressure, maximal aerobic capacity (VO_2_max), percentage of muscle mass, percentage of lower extremity muscle mass, Mini-Mental State Examination (MMSE) score, and years of education.

**Results:**

Compared with the ABW group, the SW group had significantly lower VO_2_max (14.49 ± 2.95 vs 16.25 ± 2.94 mL/kg/min) and MMSE score (25.01 ± 3.21 vs 27.35 ± 1.97), fewer years of education, and these patients were more frequently women (*P* < 0.05). In the multivariate regression models, the combination of VO_2_max, sex, and MMSE score explained only 23.5% of gait speed (*P* < 0.001). MMSE score and VO_2_max independently determined gait speed after adjustment for age, BMI, HbA1c, fasting glucose, systolic blood pressure, percent of muscle mass, percent of lower extremity muscle mass, and years of education.

**Conclusion:**

In diabetic patients without neuropathy, physical impairment and disability could be prevented by an improvement in aerobic capacity and cognitive function.

ClinicalTrials.gov number: NCT04758364

In elderly and middle-aged adults, gait performance indicates health and functional status. Gait speed at the usual pace is a strong predictor for a range of adverse outcomes and denotes the multisystemic well-being of an individual (1).

Patients with diabetes mellitus (DM) with neuropathy compared with individuals without DM have a slower walking speed, shorter step length, increased step width, prolonged stance phase, increased gait variability, and improper distribution of foot pressure (2,3). These alterations have been attributed to an impairment of sensory or motor nerves or the central nervous system and to a decreased strength of lower extremity muscles (2,4). However, impaired gait, physical capacity (5), and functional mobility tests were also found in diabetic patients without neuropathy compared with individuals without DM (3).

DM was also associated with an increased risk of cognitive deficits and dementia (6). Reduced cognitive function was identified even in early stages of DM. DM and hypertension were also separate risk factors for dementia due to the development of cerebrovascular pathologies (7).

There is a lack of studies on factors associated with gait speed in diabetic individuals without neuropathy (8). Therefore, the aim of this study was to compare possible factors affecting gait speed between slow walkers (SW) and average or brisk walkers (ABW) with DM without neuropathy. The second aim was to investigate the effect of age, sex, muscle mass, aerobic capacity, cognitive function, blood pressure, metabolic measures, and years of education on gait speed in diabetic individuals without neuropathy.

## Patients and methods

### Patients

This cross-sectional study was conducted at the Physical Medicine and Rehabilitation Clinic of Pamukkale University in February and March 2021. A total of 109 individuals with type-2 DM selected with computer-based randomization were interviewed and assessed for the presence of neuropathy. Participants self-reported a physician’s diagnosis of DM and time of onset of DM. All participants were under medical supervision and were taking anti-diabetic and/or antihypertensive agents. All could ambulate independently. We also inquired about the presence of depression and hypothyroidism, factors that also affect gait speed.

As DM duration longer than 10 years is strongly associated with the development of diabetic neuropathy, we enrolled patients with T2DM duration shorter than 10 years but longer than 1 year (5). These patients were assessed for the presence of neuropathic symptoms with the Douleur Neuropathique 4 (DN4) questionnaire. DN4 is used to assess neuropathic pain (9) and was validated for diabetic neuropathy (10). Its validity and reliability were confirmed for Turkish patients (11). Diabetic patients with scores less than 4 out of 10 points were included in the study.

Exclusion criteria were insulin therapy, poor glycemic control, manifesting cardiovascular disease, retinopathy or other visual problems, diabetic neuropathy, nephropathy, cerebrovascular disease, prominent cognitive impairment, alcohol dependence, cancer, chemo/radiotherapy, foot ulcer, orthopedic or surgical problems interfering with gait, wheelchair or any assistive devices for ambulation, or knee or hip arthritis. Eighty participants met the inclusion and exclusion criteria, and 71 accepted to participate.

Gait speed was assessed with G-walk (BTS Bioengineering, Quincy, MA, USA), a system with demonstrated validity and reliability (12) consisting of inertial sensors: a triaxial accelerometer, magnetometer, and gyroscope. It was positioned on the S1 vertebra with a semi-elastic band. The participants walked on a smooth surface for 7 m at their usual pace and returned. The cut-off point for SW was 1.0 m/s (8,13).

The factors related to both gait and DM were considered potential explanatory variables. These included age, sex, BMI, HbA1c level, fasting glucose, systolic blood pressure, maximal oxygen consumption (VO_2_max), percentage of muscle mass, percentage of lower extremity muscle mass, MMSE score, and years of education.

Height was measured without shoes on a stadiometer. Body composition was evaluated with Tanita MC580 (Tanita, Arlington Heights, IL, USA), a valid and reliable bioelectric impedance analyzer (14). Weight, body mass index, percentage of muscle mass, and percentage of lower extremity muscle mass were assessed. Muscle mass percentage was expressed with respect to body weight. Before the analyses, participants did not eat or drink for more than three hours but were prompted to urinate.

Blood glucose levels and HbA1c were detected in blood samples after overnight fasting. Blood pressure was measured with a sphygmomanometer on the left arm in the sitting position after rest.

VO_2_max was measured with the cardiopulmonary exercise test on a bicycle (Bike-med, Technogym, Cesena, Italy) by an ergometer (CareFusion 234 Gmb 2011, Hoechberg Germany) using breath-by-breath technique. Exercise testing was made by a ramp protocol starting with 30 W and increasing 15 W per minute until respiratory exchange ratio ≥1.10, when VO_2_max was measured. Blood pressure, heart rate, and ECG were monitored during resting, exercise testing, and the recovery period. Exercise tests ended without any complications.

MMSE, a questionnaire evaluating orientation, attention, calculation, memory recall, language, and visual-spatial skills (15), has been widely used for cognitive function assessment. The scores between 24 and 30 denote a normal cognitive function, scores between 18 and 23 indicate mild dementia, and scores below 17 indicate severe dementia. Its validity and reliability were confirmed for Turkish patients (16). Due to a close relationship of cognitive functions and education, education level was expressed in years.

The study was approved by the Non-invasive Clinical Research Ethics Committee of Pamukkale University. This study conformed to the Declaration of Helsinki. All participants provided written informed consent.

### Statistical analysis

Continuous variables are expressed as means ± standard deviation (SD), and categorical data are expressed as frequencies and percentages. The normality of distribution was tested with the Shapiro-Wilk test. Independent sample *t* test or Mann-Whitney U test were used to assess the differences in continuous variables between two groups. The differences in categorical variables were assessed with the χ^2^ test or Fisher exact test. The power of the study was 90%, and beta was 0.10 with respect to VO_2_max for the comparison between the groups. Multivariate linear regression models were performed to determine factors effecting gait speed. Multivariate linear regression models with a backward elimination method was performed by entering all of the independent variables into the equation first, then deleting one variable at a time if it did not contribute to the regression. *P* < 0.05 was considered significant. The analysis was performed with SPSS 17.0 software (SPSS Inc., Chicago, IL, USA).

## RESULTS

The study enrolled 71 patients (61 women) with a mean age of 55.87 ± 7.74 years (min 38-max 74). The patients’ characteristics are presented in Table 1.

**Table 1:** Characteristics of patients with type-2 diabetes mellitus (N = 71)

	Mean ± standard deviation or number (%)	(Min-Max
Age (years)	55.87 ± 7.74	38-74
Sex (male/female)	10/61 (14.1/85.9)	-
Body mass index (kg/cm^2^)	31.75 ± 4.63	
Gait speed (m/s)	1.09 ± 0.18	0.76-1.55
Hemoglobin A1c (%)	6.91 ± 0.88	5.20-10.20
Fasting glucose (mg/dL)	114.93 ± 24.20	81-212
Systolic blood pressure (mmHg)	125.86 ± 10.14	90-140
VO_2_max (kg/mL/min)	15.86 ± 3.09	
Percent of muscle mass	61.35 ± 6.45	
Percent of lower extremity muscle mass	33.69 ± 17.83	10.9-109.0
Hypertension	32 (39.5)	-
Hypothyroidism	13 (16)	-
Depression	6 (7.4)	-
Mini Mental State Examination Score	26.62 ± 2.71	18-30
Douleur Neuropathique 4 score	0.36 ± 0.12	0-1
Years of education	8.37 ± 3.72	

**Figure 1:**
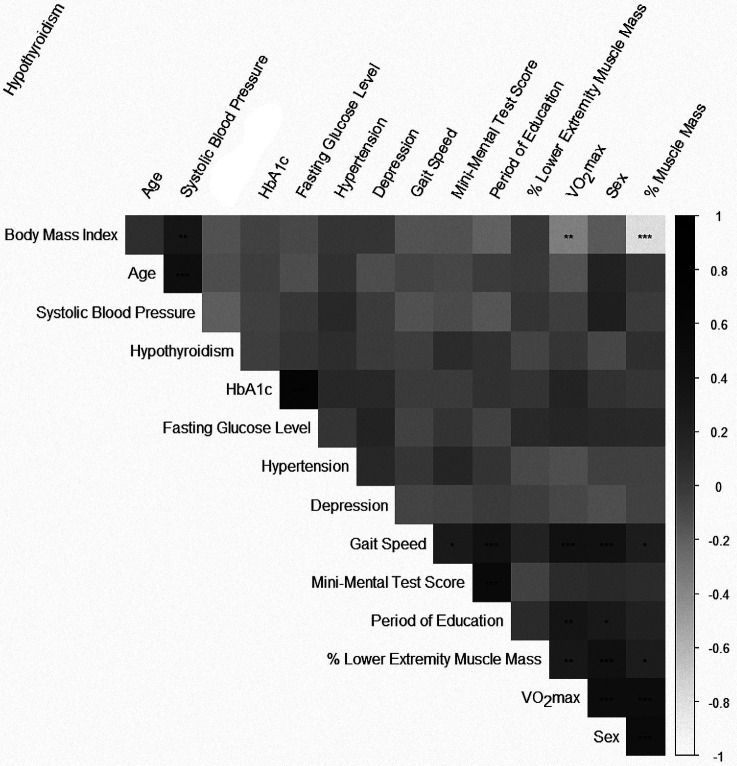
Correlation matrix for the involved parameters. Lighter tones indicate negative correlation, darker tones indicate positive correlation. *P<0.005; **P <0.001; ***P <0.0001.

The factors associated with gait speed in SW and ABW are shown in Table 2. Compared with the ABW group, the SW group had significantly lower VO_2_max (14.49 ± 2.95 vs 16.25 ± 2.94 mL/kg/min) and MMSE score (25.01 ± 3.21 vs 27.35 ± 1.97), had fewer years of education, and these patients were more frequently women (*P* < 0.05). The number of patients with depression and hypothyroidism did not significantly differ between the groups. Gait speed was related to sex, VO_2_max, muscle mass, MMSE score, and years of education (*P* < 0.05) (Figure 1).

**Table 2:** Comparison of factors associated with gait speed between slow and average/brisk walkers in patients with type-2 diabetes*

	Walkers		
	slow (n = 21)	average or brisk (n = 50)	p
Gait speed (m/s)	0.89 ± 0.65	1.18 ± 0.14	<0.001
Age (years)	56.45 ± 8.92	55.89 ± 6.74	0.723
Sex (male/female) (%)	0/21 (0/100)	10/40 (20/80)	0.027
Body mass index (kg/cm^2^)	31.90 ± 4.46	31.46 ± 4.87	0.743
Hemoglobin A1c (%)	6.88 ± 0.79	6.93 ± 0.93	0.629
Fasting glucose (mg/dL)	118.45 ± 31.61	112.98 ± 20.28	0.643
Systolic blood pressure (mmHg)	127.0 ± 8.01	124.56 ± 10	0.556
Maximal aerobic capacity (kg/mL/min)	14.49 ± 2.95	16.25 ± 2.94	0.029
Percentage of muscle mass	60.71 ± 5.28	61.75 ± 6.99	0.845
Percentage of lower extremity muscle mass	31.37 ± 0.74	34.67 ± 21.23	0.629
Mini Mental State Examination Score	25.01 ± 3.21	27.35 ± 1.97	0.040
Year of education (years)	5.39 ± 3.73	9.54 ± 3.14	0.010
Comorbid diseases			
hypertension	9 (42.9)	23 (46)	0.808
hypothyroidism	5 (23.8)	8 (16.0)	0.437
depression	1 (4.8)	5 (10)	0.469
Pharmacological therapies	**(user/non-user)**	**(user/non-user)**	
metformin	20/1 (95.2/4.8)	46/4 (92/8)	0.999
dipeptidyl peptidase-4 inhibitors	6/15 (28.6/71.4)	18/32 (36/64)	0.546
sulphonylureas	3/18 (14.3/85.7)	8/42 (16.0/84)	0.855
SGLT2 inhibitors	2/19 (9.5/90.5)	7 /43(14.0/86)	0.716
angiotensin receptor blockers	6/15 (28.6/71.4)	11/39 (22.0/78)	0.554
calcium channel blockers	1/20 (4.8/95.2)	9/41 (18/82)	0.262
Beta blockers	4/17 (19/81)	3/47 (6/94)	0.184
diuretics	1/20 (4.8/95.2)	3/47 (6/94)	0.999
angiotensin converting enzyme inhibitors	2/19 (9.5/90.5)	5/45 (10/90)	0.999

*data are presented as mean ± standard deviation or number (%).

A series of multifactorial linear regression models was performed to examine the relationship between multiple factors and gait speed (R) and assess how these factors potentially explained gait speed (R^2^). Sex, age, and years of education were included in the models as confounding factors (Table 3). Adjusted R^2^ was used to eliminate the effect of several variables on R^2^. Model 1 included VO_2_max_,_ sex, MMSE score, age, body mass index, fasting glucose, HbA1c, systolic blood pressure, percentage of muscle mass, percentage of muscle mass of lower extremities, and years of education (*P* < 0.05). All the models had a significant effect on gait speed. Significance progressively increased with each model, and Model 10, which included VO_2_max, sex, and MMSE score, attained the lowest *P* value. VO_2_max and MMSE score significantly positively correlated with gait speed after adjustment for age, BMI, HbA1c, fasting glucose, systolic blood pressure, percentage of muscle mass, percentage of lower extremity muscle mass, and years of education (Table 4).

**Table 3:** Multivariate linear regression models of each factor associated with gait speed corrected for age, sex, and education years for patients with type-2 diabetes mellitus

Factors	B (Standard Error)	Standardized Beta	p factor	R	Adjusted R^2^	p model	95% confidence interval	Variance inflation factor
Body mass index (kg/cm^2^)	-0.002 (0.005)	-0.048	0.671	0.519	0.269	0.01	-0.11 -0.007	1.080
Hemoglobin A1c	-0.005 (0.024)	-0.022	0.761	0.489	0.189	0.02	-0.53-0.43	1.010
Fasting glucose	-0.001(0.001)	-0.084	0.459	0.522	0.224	0.01	-0.002- 0.001	1.047
Systolic blood pressure	-0.003(0.002)	-0.136	0.281	0.533	0.284	0.01	-0.007- 0.002	1.334
Maximal aerobic capacity	0.012 (0.008)	0.192	0.158	0.542	0.294	0.01	-0.005-0.028	1.559
Percent of muscle mass	0.002 (0.004)	0.061	0.642	0.519	0.269	0.01	-0.006-0.009	1.429
Hypothyroidism	-0.010 (0.054)	-0.021	0.855	0.418	0.124	0.01	-0.117-0.098	1.034
Depression	-0.023 (0.075)	-0.034	0.736	0.419	0.125	0.01	-0.171- 0.126	1.026
Mini Mental State Examination score	0.007 (0.009)	0.099	0.451	0.523	0.227	0.01	-0.011- 0.24	1.446
Percent of lower extremity muscle mass	0 (0.001)	-0.001	0.995	0.517	0.220	0.01	-0.002- 0.002	1.120

**Table 4:** Highly significant multivariate linear regression models with predictive factors for gait speed in patients with type-2 diabetes mellitus

Factors	B (Standard Error)	Standardized Beta	p factor	R	Adjusted R^2^	p model	95% confidence interval	Variance inflation factor
**Model 8**				0.551	0.241	0.01		
Maximal aerobic capacity	0.015 (0.008)	0.242	0.069				-0.01-0.031	1.368
Sex	-0.005 (0.024)	-0.022	0.027				0.18-0.297	1.382
Mini Mental State Examination score	-0.001(0.001)	-0.084	0.059				-0.001-0.032	1.025
Fasting glucose	-0.003(0.002)	-0.136	0.326				-0.003-0.001	1.018
Systolic blood pressure	0.012 (0.008)	0.192	0.221				-0.007- 0.020	1.119
**Model 9**				0.539	0.241	0.001		
Maximal aerobic capacity	0.014 (0.008)	0.233	0.079				-0.002-0.030	1.360
Sex	0.154 (0.069)	0.291	0.030				-0.015-0.294	1.379
Mini Mental State Examination score	0.016 (0.008)	0.223	0.053				(0-0.032	1.022
Systolic blood pressure	-0.010 (0.054)	-0.021	0.855				-0.07-0.002	1.117
**Model 10**				0.523	0.235	0.0003		
Maximal aerobic capacity	0.017 (0.008)	0.272	0.036				0.001-0.032	1.274
Sex	0.131 (0.067)	0.248	0.055				-0.03-0.266	1.274
Mini Mental State Examination score	0.017 (0.008)	0.239	0.038				0.001-0.033	1.008

## DISCUSSION

In this study, the SW group had significantly lower VO_2_max and MMSE score, fewer years of education, and the patients were more frequently women. However, the combination of VO_2_max, sex, and MMSE score explained only 23.5% of gait speed. VO_2_max, and MMSE scores were mutually positively correlated and significantly contributed to gait speed.

Older adults are known to have a slower gait speed (4,17). Older adults with T2DM have decreased stride length and increased gait variability, particularly during dual-task conditions irrespective of the neuropathy status (18). Similar results were reported in middle-aged patients with diabetes (19). Our study involved mostly middle-aged patients, while other studies involved elderly or frail people, which might have obscured the effect of age on gait speed. In our study, age did not differ between SW and ABW, but it was included in regression models due to its importance in the literature.

Yavuzer et al showed that diabetic individuals without neuropathy and non-diabetic individuals significantly differed in the speed of gait and length of steps, indicating that gait alteration can be encountered in individuals without neuropathy. Most of the population-based studies also did not take into account the neuropathy status of diabetic patients. One study showed that older women with DM duration of more than 10 years had a slower gait speed and smaller step length compared with women with DM duration of less than 10 years (8). To exclude the effects of diabetic neuropathy, our study involved participants who had DM for less than 10 years, and 29.6% of them were SW.

Slow gait speed independently predicted MMSE score decline during seven years of follow-up (20). It also predicted the onset of dementia, Alzheimer’s disease, or an increased cognitive decline (1). Although our participants had mild cognitive impairments, SW had significantly lower MMSE scores. Additionally, MMSE score was one of the independent determinants of gait speed. In another study, gait speed was the only independent determinant of mild cognitive impairment in patients with DM (13). DM impairs psychomotor speed and processing, visual-spatial abilities, learning, memory, executive functioning, and attention (18). In diabetic individuals with and without neuropathy, dual-task conditions during gait reduced gait performance (18). In another study, derangements in cognition and gait were interrelated and common in individuals with DM and/or hypertension (21). Furthermore, non-demented older adults with hypertension (22) and non-demented older adults with DM (21) had a decreased cognitive performance. In our study, the MMSE scores were adjusted for several confounding factors including systolic blood pressure and metabolic factors.

The mean resting systolic blood pressure in this study was 125.86 ± 10.14 mm Hg while the participants were on antihypertensive agents. Systolic blood pressure values did not differ between SW and ABW, and systolic blood pressure contributed non-significantly to all models except Model 10. This might be explained by a close-to-normal range of blood pressure in our patients. In other studies, hypertensive older adults had a slower gait speed than normotensive older patients (23,24).

In our study, SW and ABW did not differ in either HbA1c or fasting glucose levels. These parameters also did not contribute significantly to the models. The literature results on the relationship between gait and HbA1c are inconclusive. Lower HbA1c and blood glucose levels were related to brisk walking pace (25,26). However, the Rotterdam study found no relation between impaired fasting glucose and continuous glucose levels during gait (19). In another study, HbA1c level was not related to knee extensor strength and gait speed after adjustment for body weight (17). In contrast, higher HbA1c levels were related to a worse physical, but not cognitive function, after adjustment for several factors (27). A population-based study found that HbA1c levels of >8% were related to a slower gait (28). Tight glucose control regimes might cause hypoglycemic episodes leading to impaired cognition. Higher blood glucose levels, on the other hand, could also cause neuropathy or impaired cognition by leading to structural changes in the brain (28). In this study, neither HbA1c nor fasting blood glucose were correlated with any other factor. Fasting blood glucose was not below 70 mg/dL in any of the participants, thus hypoglycemia could not have been the factor affecting gait.

VO_2_max is a measure of cardiac, pulmonary, and muscular functioning. Despite its well-known relation with gait speed, it has not been included in most of the population-based studies. Therefore, direct measurement of aerobic capacity is a strength of this study. In the present study, VO_2_max strongly predicted gait speed and was related to the percentage of muscle mass and lower extremity muscle mass. In other studies, VO_2_max was associated with most of the self-selected walking speed options when corrected for age, weight, height, and obesity (29). Other studies showed that individuals with T2DM had lower aerobic exercise capacity compared with healthy controls (30). Another regression model that included leg strength, VO_2_max_,_ weight, height, and muscle strength predicted 26% of gait speed (31).

We used the percentage of muscle mass as the muscle mass of the body was corrected by weight. The percentage of muscle mass and the percentage of lower extremity muscle mass did not significantly differ between SW and ABW. Although the percentage of muscle mass was associated with gait speed, BMI, sex, and VO_2_max, after adjustment it did not significantly contribute to gait speed. In some clinical and population-based studies, T2DM was related to loss in muscle mass and strength (17,22). Diabetic neuropathy might cause a loss of motor neurons and thus muscle mass. Diabetic patients over 65 years had lower muscle density, knee and ankle muscle strength, muscle power and quality, and slower gait compared with non-diabetic individuals (32). They also had decreased quadriceps muscle power, strength, and gait speed. Muscular strength loss was faster in people who had diabetes for over 3 years (17). Longer disease duration (>6 years) and poor glycemic control (HbA1c >8.0%) were related to a low muscle quality (33). Muscle quality was significantly lower in the arms or legs of diabetic patients compared with non-diabetic people (17).

This study suffers from several limitations. The fitness level and gait speed follow a nonlinear relation (31), which cannot be sufficiently explained by linear regression models. Second, due to a limited number of participants, men and women were not equally distributed across SW and ABW groups. This might have affected the significant contribution of sex in the models.

In conclusion, gait is a highly integrated function of multiple coordinated physiological systems, all of which are progressively impaired by DM. This study provides important information about alterations in gait in diabetic patients without neuropathy. In these patients, physical impairment and disability could be prevented by an improvement in aerobic capacity and cognitive function.
